# Aberrant Resting-State Functional Connectivity of the Dorsal Attention Network in Tinnitus

**DOI:** 10.1155/2021/2804533

**Published:** 2021-12-31

**Authors:** Haimeng Hu, Yining Lyu, Shihong Li, Zheng Yuan, Chuntao Ye, Zhao Han, Guangwu Lin

**Affiliations:** ^1^Department of Radiology, Huadong Hospital, Fudan University, Shanghai, China; ^2^Department of Otorhinolaryngology, Huadong Hospital, Fudan University, Shanghai, China

## Abstract

Previous functional magnetic resonance imaging (fMRI) analyses have shown that the dorsal attention network (DAN) is involved in the pathophysiological changes of tinnitus, but few relevant studies have been conducted, and the conclusions to date are not uniform. The purpose of this research was to test whether there is a change in intrinsic functional connectivity (FC) patterns between the DAN and other brain regions in tinnitus patients. Thirty-one patients with persistent tinnitus and thirty-three healthy controls were enrolled in this study. A group independent component analysis (ICA), degree centrality (DC) analysis, and seed-based FC analysis were conducted. In the group ICA, the tinnitus patients showed increased connectivity in the left superior parietal gyrus in the DAN compared to the healthy controls. Compared with the healthy controls, the tinnitus patients showed increased DC in the left inferior parietal gyrus and decreased DC in the left precuneus within the DAN. The clusters within the DAN with significant differences in the ICA or DC analysis between the tinnitus patients and the healthy controls were selected as regions of interest (ROIs) for seeds. The tinnitus patients exhibited significantly increased FC from the left superior parietal gyrus to several brain regions, including the left inferior parietal gyrus, the left superior marginal gyrus, and the right superior frontal gyrus, and decreased FC to the right anterior cingulate cortex. The tinnitus patients exhibited decreased FC from the left precuneus to the left inferior occipital gyrus, left calcarine cortex, and left superior frontal gyrus compared with the healthy controls. The findings of this study show that compared with healthy controls, tinnitus patients have altered functional connections not only within the DAN but also between the DAN and other brain regions. These results suggest that it may be possible to improve the disturbance and influence of tinnitus by regulating the DAN.

## 1. Introduction

Tinnitus refers to the continuous and conscious abnormal perception of nonverbal sounds in the ear or brain in the absence of acoustic stimulation. Thus, it is a subjective feeling [1–3]. Tinnitus occurs at all ages and affects 10-25% of people worldwide [1, 4]. Patients with persistent tinnitus also experience problems such as anxiety, hearing loss, insomnia, depression, decreased attention, and cognitive impairment, which have some impact on their lives [5, 6].

To date, there is no final conclusion regarding the mechanism underlying tinnitus, but as a result of in-depth studies, it is widely believed that sustained tinnitus involves multisystem participation and impact [1, 6, 7]. Based on this theory, emotion, attention, memory, and control networks all contribute to the abnormal sound perception of tinnitus and the generation and development of related features. Kapolowicz and Thompson [8, 9] found that tinnitus may be closely related to changes in the plasticity of the central nervous system. Their research showed that plasticity changes in the central auditory system are caused by unevenness in auditory neuronal excitability and inhibition. When this unequal input exceeds its adjustment range, it abnormally stimulates neurological activity in multiple nervous systems, including nerve synchronization [10, 11]. Therefore, the study of the nonauditory system related to tinnitus has also received attention.

Previous electrophysiological studies [12] have shown that tinnitus patients exhibit different neurological activities and neural connections compared with normal controls. Many studies based on functional magnetic resonance imaging (fMRI) [13–16] have also reported changes in the nervous system associated with tinnitus. Previously, some researchers thought that tinnitus may be a result of the maladaptation of the auditory nervous system [17]. However, studies based on fMRI [6, 18] have reported that the auditory neural networks of patients with tinnitus do not significantly change. This finding suggests that the neurological activity of the auditory network may not play a role in tinnitus. Several studies [19] have found that in the neural networks of nonauditory areas, such as the default mode network (DMN), the dorsal attention network (DAN), and the limbic system, tinnitus patients show abnormal functional connection changes. However, whether this change in neural function is the cause of tinnitus or its consequence has not been determined. The DAN mainly includes the bilateral parietal lobe and frontal visual area, which are responsible for top-down attention orientation and participate in exogenous attention orientation [20, 21]. Tinnitus patients may experience changes in the function of the DAN as they pay attention to and overcome tinnitus and hallucinations, but thus far, few related studies have been conducted, and their conclusions differ. Husain et al. [22] found that activation of the DAN in patients with tinnitus was significantly decreased. However, Schmidt et al.'s research [23] showed that tinnitus patients had increased neurofunctional connections in the DAN, which was significantly related to the severity of tinnitus. Therefore, we applied fMRI and used the analytical methods of an independent component analysis (ICA), a degree centrality (DC) analysis, and a seed-based functional connectivity (FC) analysis to explore the functional connections between the DAN and other brain regions in tinnitus patients from multiple perspectives to further explore the possible role of the DAN in the occurrence and development of tinnitus.

## 2. Materials and Methods

### 2.1. Participants

Thirty-one patients (twenty-four males, seven females, mean age 38.58 years) with persistent tinnitus and thirty-three healthy controls (twenty-three males, ten females, mean age 38.24 years) were enrolled in this cross-sectional study. The healthy controls were matched with the patients by age and sex. All subjects were right-handed and aged between 18 and 75 years. Patients with rhythmic tinnitus such as vascular tinnitus and those with otitis or periauricular neoplastic diseases were excluded. Subjects with contraindications for magnetic resonance imaging (MRI), disabling hearing impairment (hearing thresholds > 40 dB, hearing level at 0.5, 1, 2, and 4 kHz) [24], alcohol or drug abuse, family history of mental disorders or hereditary neurological diseases, and organic lesions on routine MRI scans were excluded. All patients completed the Tinnitus Handicap Inventory (THI) to evaluate the severity of tinnitus before undergoing MRI. The THI consists of 25 items with a maximum score of 100 points. Higher scores indicate more severe tinnitus. According to the THI score, the severity of tinnitus is divided into 5 grades as follows [25]: grade I (slight, THI 0-16), grade II (mild, THI 18-36), grade III (moderate, THI 38-56), grade IV (severe, THI 58-76), and grade V (catastrophic, THI 78-100). The experiment was approved by the ethics committee (Shanghai Huadong Hospital, Fudan University, 2020K135). All subjects signed informed consent forms.

### 2.2. Imaging Acquisition

In this study, all MRI images were acquired on a 3.0 T magnetic resonance scanner (Magnetom Prisma, Siemens Healthineers) using a 20-channel phased-array head coil. During the MRI scan, head fixation was performed to prevent head movement and a cotton plug was used to reduce noise effects. The patients were required to stay still, close their eyes, stay awake, and avoid any thinking activity to the greatest extent possible. First, routine MRI sequences (T1-weighted imaging (T1WI), T2-weighted imaging (T2WI), diffusion-weighted imaging (DWI), and T2-weighted fluid attenuated inversion recovery (T2-FLAIR)) of the head were carried out, and after a senior imaging diagnostic physician determined that there was no obvious abnormality in the brain parenchyma, the next sequence scan of structural and functional images was carried out. Structural images were obtained by a T1WI 3-dimensional magnetization prepared rapid acquisition gradient echo (T1WI-3D-MPRAGE) sequence with the following parameters: repetition time (TR) = 2300 ms, echo time (TE) = 2.32 ms, slice thickness = 0.9 mm, acquisition matrix = 256 × 256, and flip angle = 8°. In total, 192 images were obtained. Resting-state fMRI (rs-fMRI) images were obtained by a multilayer simultaneous acquisition technique (simultaneous multislice, SMS) with the following parameters: TR = 2500 ms, TE = 30 ms, slice thickness = 1.5 mm, slices = 72, in‐plane voxel size = 2 × 2 mm^2^, field of view (FOV) read = 192 mm × 192 mm, FOV phase = 100%, and flip angle = 80°. In total, 8640 images were obtained. Each resting-state measurement contained 120 volumes, resulting in a total acquisition time of 5.15 minutes. All image sequences were carried out by the same senior technician at our department.

### 2.3. Image Processing

#### 2.3.1. Image Preprocessing

The Graph-theoretical Network Analysis Toolkit (GRETNA 2.0; http://www.nitrc.org/projects/gretna/) toolbox [26] and the Statistical Parametric Mapping 12 (SPM12, http://www.fil.ion.ucl.ac.uk/spm) software package were used to preprocess the rs-fMRI images. First, the first five scans were removed to prevent signal interference that may be caused by machine instability. Then, the data were corrected by slice timing and realigned to eliminate errors due to acquisition time differences or head movement. After standardization and resampling with a voxel size of 3 mm × 3 mm × 3 mm, the normalized images were smoothed by isotropic Gaussian check with a 6 mm full width at half maximum (FWHM) to render the data more consistent with the Gaussian field model and facilitate statistical inference. After the head motion analysis, the subjects whose translational movement was greater than 2 mm or more than 2° rotation were excluded from further statistical analysis.

#### 2.3.2. Independent Component Analysis (ICA)

**Figure 1:**
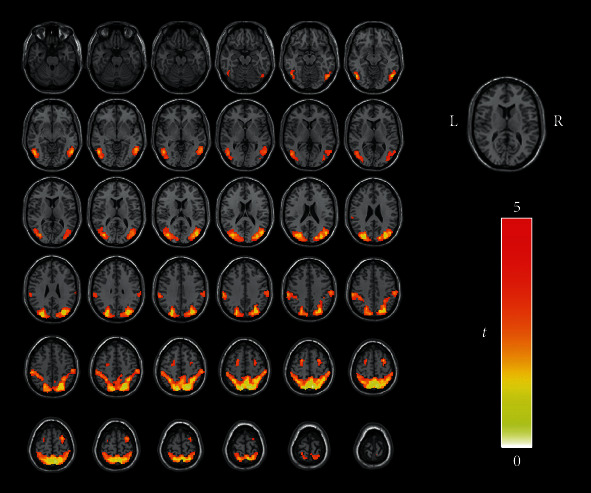
All subjects underwent single-sample *t*-tests of the DAN component obtained from ICA (FWE-corrected *P* < 0.001). The color bar indicates the *t* value of single-sample *t*-tests of two groups. Acronyms: DAN: dorsal attention network; ICA: independent component analysis; FWE: familywise error; L: left; R: right.

The group ICA was performed using Group ICA of fMRI Toolbox V4.0a (GIFT V4.0a, http://mialab.mrn.org/software/gift). The two groups were analyzed together using the preprocessed data of each subject. According to the minimum description length (MDL), the number of independent components was set to 15 [27]. The ICA was carried out by the infomax algorithm and resulted in 15 independent spatial distribution activation diagrams and corresponding time series. Among the 15 components identified, the one most similar to the DAN was visually selected. To support this selection, we applied the best spatial matching algorithm to identify the DAN, and the result was consistent with the previous method. The DAN template was downloaded from GIFT's website (http://mialab.mrn.org). A one-sample *t*-test was used to derive the DAN FC maps of the two groups using SPM12 (familywise error, FWE-corrected *P* < 0.001) (Figure 1). Then, we used this result as the mask of the DAN.

#### 2.3.3. Degree Centrality (DC) Analysis

DC is a voxelwise measurement that mainly calculates the strength of functional connection between a voxel and all other voxels in the whole brain. DC measures the importance of a given voxel in the entire brain region by describing the ability of a single voxel to transmit information to other brain regions. Continuous preprocessing before the DC calculation was performed using GRETNA V2.0. Linear detrending and a temporal filter (0.01-0.08 Hz) were applied to the unsmoothed preprocessed image to reduce low-frequency drifts and high-frequency physiological noise. Then, irrelevant signals, such as white matter signals, cerebrospinal fluid signals, and the Friston 24-parameter model head motion parameters, were regressed out of the time series of each voxel. The weighted DC calculation was performed using the Resting-State fMRI Data Analysis Toolkit plus V1.2 (REST plus V1.2, http://www.restfmri.net/forum). We calculated the DC of the voxels in the whole brain with Pearson correlation coefficients greater than 0.25 and transformed them into a *z*-score map using Fisher's *z* transformation. Subsequently, all *z*-score maps were spatially smoothed (FWHM = 6 mm × 6 mm × 6 mm).

#### 2.3.4. Seed-Based Functional Connectivity (FC) Analysis

The clusters within the DAN with significant differences in the ICA (refer to Sections 2.4 and 3.2) or DC analysis (refer to Sections 2.4 and 3.3) between the tinnitus group and the healthy control group were selected as regions of interest (ROIs) for seeds. The smoothed preprocessed images were subjected to detrending, bandpass filtering, and nuisance covariance regression as described in Section 2.3.3. Then, we calculated the correlations between the ROI and each voxel within the brain in each subject and converted them into a *z*-score map to improve normality using REST plus V1.2.

### 2.4. Statistical Analysis

A chi-square test was used to assess the differences in the categorical variables (sex and education level) between the two groups. Age was compared using an independent two-sample *t*-test. The statistical significance of the differences between the groups was set to a *P* value < 0.05. The statistical analysis was performed using SPSS version 17.0 (IBM, USA).

For the ICA, independent two-sample *t*-tests were used to test the group difference in FC within the DAN using the DAN FC map (described in Section 2.3.2) as the mask. To analyze the DC differences within the DAN, we performed independent two-sample *t*-tests between the tinnitus patients and healthy controls using the DAN template as the mask. In the seed-based FC analysis, we used independent two-sample *t*-tests and the gray matter of the whole brain as a mask to test the group difference in FC. All these voxelwise analyses were performed using SPM12. All results are presented at the statistical threshold of uncorrected voxelwise *P* < 0.001 combined with an FWE-corrected clusterwise threshold of *P* < 0.05 [28].

## 3. Results

### 3.1. Demographic and Clinical Characteristics

In this study, 31 patients with persistent tinnitus and 33 healthy controls were included. The demographic and clinical data of the two groups are shown in Table 1. There was no significant difference in age (two-sample *t*-test, *P* = 0.917), sex (Pearson chi-square test, *P* = 0.485), or education level (chi-square test, *P* = 0.370) between the two groups. The mean THI score of the tinnitus patients was 46.26 ± 26.26. According to the THI score, the tinnitus patients had different levels of tinnitus severity. There were 6 patients in grade I, 9 patients in grade II, 4 patients in grade III, 7 patients in grade IV, and 5 patients in grade V. The mean tinnitus duration was 32.68 ± 55.54 months, and there were 4 patients with acute tinnitus (less than 3 months), 4 patients with subacute tinnitus (over 3 months but less than 6 months), and 23 patients with chronic tinnitus (6 months or longer).

### 3.2. ICA

**Figure 2:**
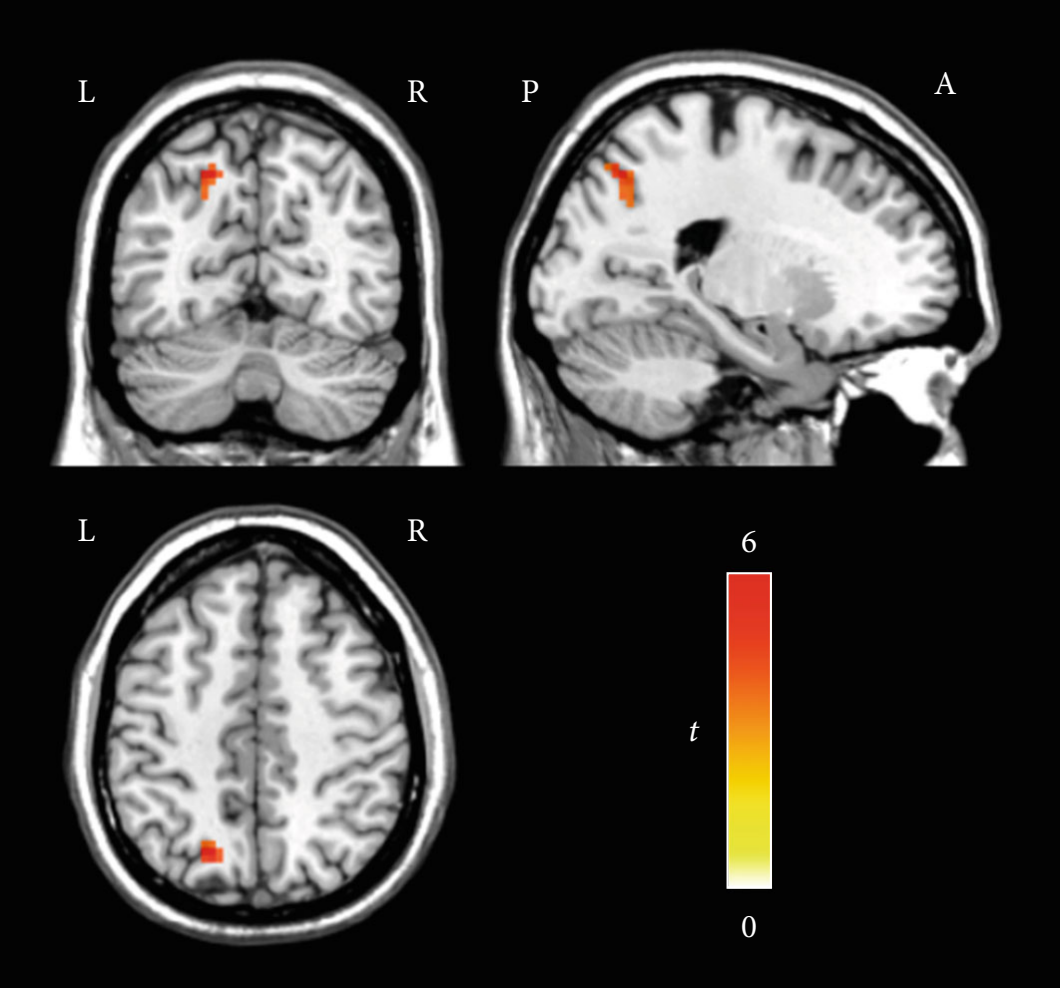
Regions showing increased FC in the DAN in tinnitus patients (voxelwise *P* < 0.001, FWE-corrected clusterwise *P* < 0.05). The tinnitus patients showed increased connectivity in the left superior parietal gyrus in the DAN compared to the healthy controls. The color bar indicates the *t* value of independent two-sample *t*-tests between the groups. Acronyms: DAN: dorsal attention network; FC: functional connectivity; FWE: familywise error; L: left; R: right: A: anterior; P: posterior.

A group ICA was applied to observe the difference in functional connections within the DAN between the two groups. Using the DAN FC maps as the mask, the tinnitus patients showed increased connectivity in the left superior parietal gyrus (voxels = 24, *T* = 5.817, *x* = −18, *y* = −66, *z* = 48, voxelwise *P* < 0.001 combined with an FWE-corrected clusterwise *P* < 0.05) in the DAN compared to the healthy controls, as shown in Figure 2.

### 3.3. DC Analysis

**Figure 3:**
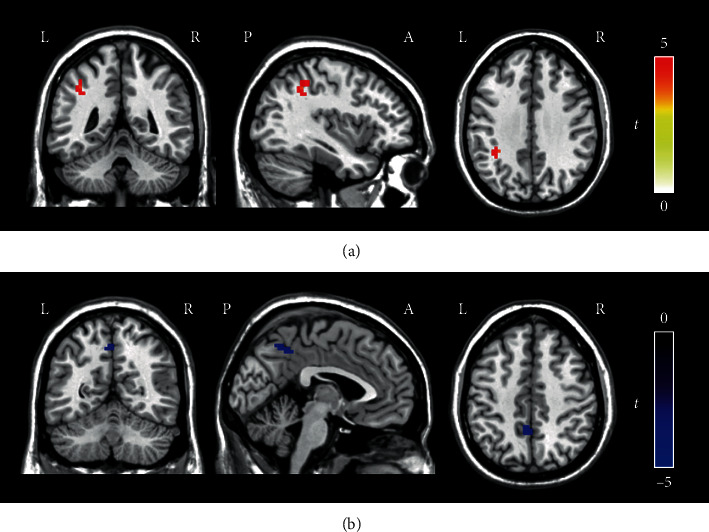
Regions showing different DC in the DAN between the tinnitus patients and healthy controls (voxelwise *P* < 0.001, FWE-corrected clusterwise *P* < 0.05). (a) The tinnitus patients showed increased DC in the left inferior parietal gyrus in the DAN compared to the healthy controls. (b) The tinnitus patients showed decreased DC in the left precuneus in the DAN compared to the healthy controls. The color bar indicates the *t* value of independent two-sample *t*-tests between the groups. Red represents increased DC in the tinnitus patients compared with the healthy controls, while blue shows decreased DC. Acronyms: DAN: dorsal attention network; DC: degree centrality; FWE: familywise error; L: left; R: right; A: anterior; P: posterior.

Compared with the healthy controls, the tinnitus patients showed increased DC in the left inferior parietal gyrus (voxel = 32, *T* = 4.344, *x* = −39, *y* = −42, *z* = 48, voxelwise *P* < 0.001 combined with an FWE-corrected clusterwise *P* < 0.05) and decreased DC in the left precuneus (voxel = 25, *T* = −4.325, *x* = −6, *y* = −45, *z* = 45, voxelwise *P* < 0.001 combined with an FWE-corrected clusterwise *P* < 0.05) within the DAN (Figure 3).

### 3.4. Seed-Based FC Analysis

**Figure 4:**
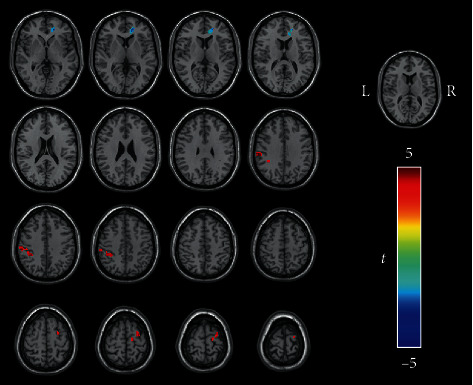
Regions showing different FC in the tinnitus patients compared with the healthy controls when the left superior parietal cortex was used as the seed (voxelwise *P* < 0.001, FWE-corrected clusterwise *P* < 0.05). The tinnitus patients exhibited significantly increased FC from the left superior parietal gyrus to the left inferior parietal gyrus, the left superior marginal gyrus, and the right superior frontal gyrus but showed decreased FC to the right anterior cingulate cortex. The color bar indicates the *t* value of independent two-sample *t*-tests between the groups. Red represents regions with increased FC with seeds in the tinnitus patients compared with the healthy controls, while blue shows regions with decreased FC. Acronyms: DAN: dorsal attention network; FWE: familywise error; FC: functional connectivity; L: left; R: right.

Based on the ICA results, the left superior parietal gyrus was defined as a seed to analyze the differences in the brain networks between the two groups. Compared with the healthy controls, the tinnitus patients exhibited significantly increased FC from the left superior parietal gyrus to several brain regions, including the left inferior parietal gyrus/the left superior marginal gyrus (voxel = 88, *T* = 5.329, *x* = −60, *y* = −24, *z* = 36, voxelwise *P* < 0.001 combined with an FWE-corrected clusterwise *P* < 0.05) and the right superior frontal gyrus (voxel = 52, *T* = 4.437, *x* = 27, *y* = −6, *z* = 66, voxelwise *P* < 0.001 combined with an FWE-corrected clusterwise *P* < 0.05) and decreased FC to the right anterior cingulate cortex (ACC) (voxel = 58, *T* = −5.436, *x* = 15, *y* = 42, *z* = 0, voxelwise *P* < 0.001 combined with an FWE-corrected clusterwise *P* < 0.05) (Figure 4).

**Figure 5:**
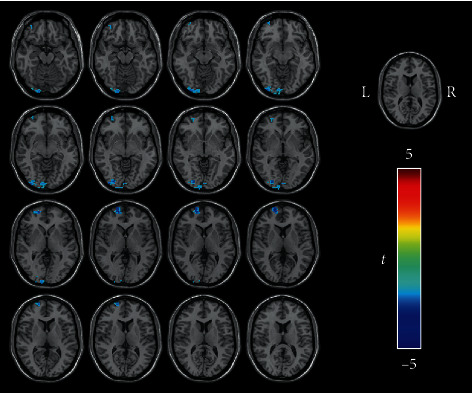
Regions showing decreased FC in the tinnitus patients when the left precuneus was used as the seed (voxelwise *P* < 0.001, FWE-corrected clusterwise *P* < 0.05). In the tinnitus patients, the left inferior occipital gyrus, left calcarine cortex, and left superior frontal gyrus showed decreased FC with the left precuneus compared with those in the healthy controls. The color bar indicates the *t* value of independent two-sample *t*-tests between the groups. DAN: dorsal attention network; FC: functional connectivity; FWE: familywise error; L: left; R: right.

Two clusters with significantly different DCs between the two groups were selected as seeds to detect differences in the brain networks via a seed-based FC analysis. In the tinnitus group, the left inferior occipital gyrus/left calcarine cortex (voxel = 145, *T* = −4.229, *x* = −30, *y* = −87, *z* = −6, voxelwise *P* < 0.001 combined with an FWE-corrected clusterwise *P* < 0.05) and left superior frontal gyrus (voxel = 78, *T* = −4.560, *x* = −21, *y* = 66, *z* = 6, voxelwise *P* < 0.001 combined with an FWE-corrected clusterwise *P* < 0.05) showed decreased FC with the left precuneus compared with those in the healthy controls (Figure 5). When the left inferior parietal gyrus was used as the seed, there was no significant difference in FC between the two groups.

## 4. Discussion

In this study, rs-fMRI was used to examine the differences in the neural network of the DAN between the tinnitus group and the healthy control group. Group ICA, DC analysis, and seed-based FC analysis were performed and revealed differences between the tinnitus patients and the healthy controls in the intrinsic FC pattern of the DAN. We also found that some regions of the DAN in the tinnitus patients differed in their contributions to the neurological activity of the whole brain, and the functional connections between these regions and some other brain regions were also altered.

By analyzing the functional connection maps of the DAN in the two groups, we found that the connectivity of the left superior parietal gyrus was enhanced in the tinnitus group, indicating that this cluster in the DAN was more activated in the tinnitus patients than in the healthy controls. The superior parietal gyrus performs complex functions; for example, it participates in the spontaneous regulation of attention and other functions [29]. The study by Mantini et al. [30] also revealed that activity in the superior parietal gyrus was increased during the resting state. They thought that this was related to brain activity, suggesting that the brain's attention system roams during the resting state. Therefore, the activation of this neural activity in the left superior parietal gyrus may indicate that the attention system in patients with tinnitus may be more active at rest than that in the control group.

When using the left superior parietal gyrus as the seed region in the whole brain FC analysis, we found that in the tinnitus patients, the left inferior parietal gyrus, the left superior marginal gyrus, and the right superior frontal gyrus showed increased FC with the left superior parietal gyrus compared with those in the healthy controls. Both the left inferior parietal gyrus and the left superior marginal gyrus belong to the inferior parietal lobule and participate in the functional adjustment of the attention network. The study by Schmidt et al. [31] also revealed changes in the functional connection of the left inferior parietal gyrus and the right superior marginal gyrus. The superior frontal gyrus belongs to the frontal eye field (FEF) and plays an important role in visual attention. Braga et al. [32] found that the FEF is involved in auditory attention. Lan et al. [33] and Shahsavarani et al. [6] found changes in the functional connections of the superior frontal gyrus in patients with tinnitus. Our findings show that the FC of several brain regions within the DAN was increased and likely confirm the conjecture that tinnitus, visual attention, and auditory attention influence each other, echoing Shahsavarani et al.'s [6] study.

Compared with the healthy controls, the FC between the left superior parietal gyrus and the right ACC was decreased in the tinnitus patients. The ACC belongs to the limbic system and is a core structure of the limbic system [5, 34]. The ACC is related to feelings, reactions, the expression of emotion, memory (especially memory related to emotion), etc. [34, 35]. Previous studies [16, 36, 37] have reported the effect of the limbic system on tinnitus. These studies showed that limbic system damage may play a role in the occurrence of tinnitus. Kapolowicz and Thompson et al. [9] believe that the abnormal function of the limbic system may appear in the early stage of tinnitus and persist during tinnitus. For a long time, the negative impact of tinnitus has been noted and connected to the limbic system [38]. Our results show that the FC between the DAN and limbic system was increased in the tinnitus patients. This finding may be related to the negative emotions caused by the patients' attention to an annoying sound.

We found that the DC of the left inferior parietal gyrus was significantly increased in the tinnitus patients, while that of the left precuneus within the DAN was suppressed. The inferior parietal gyrus plays an important role in the attention network. The function of the inferior parietal gyrus is complex and varied [39]. For example, it participates in the regulation of sensory functions, cognitive functions, language, and other functions and is closely related to somatosensory sensation. Shulman et al. [40] found that the left inferior parietal gyrus was activated by auditory stimulation. Tinnitus patients always feel that they are in an abnormal sound environment, even at rest. Consequently, they cannot avoid auditory stimulation. Therefore, we think that the increase in the functional activity of the left inferior parietal gyrus may be related to the fact that patients with tinnitus are continuously forced to experience hallucinated auditory stimulation of tinnitus. It may be the result of the patients' perception of the tinnitus sound and their attempt to understand and recognize its meaning or may be the cause of tinnitus. The specific causality needs to be further studied.

The precuneus, an important node of the attention system, participates in the regulation of many high-level cognitive functions, including the processing of self-related information, cognition, and awareness. Previous studies [30, 41, 42] have shown that the functional activity of the precuneus was decreased in normal people at rest; thus, in the resting state, it is unnecessary for people to devote much neural activity to high-level cognition. Our study was similar to that by Shahsavarani et al. [6] and Schmidt et al. [23]. We found that the functional activity of the precuneus in the tinnitus patients was lower than that in the healthy control group. We speculate that this change indicates that tinnitus patients have a reduced collection of tinnitus sound information. This change may help reduce the perception of tinnitus, which can reduce unnecessary information processing and distract attention from auditory stimuli and may be related to tinnitus patients' efforts to overcome and adapt to tinnitus.

The changes in the functional activity of the left inferior parietal gyrus and precuneus in patients with tinnitus indicate the participation of the attention system in tinnitus. On the one hand, since the sound stimulation of tinnitus is continuous, the attention system must accept and cope with this stimulus; this acceptance is characterized by an increase in the DC of the left inferior parietal gyrus. On the other hand, to cope with this disturbing auditory stimulation, the neural activity of the relevant brain regions is weakened or suppressed, which manifests as a decrease in the DC in the left precuneus. This change may help reduce the effects of or help the brain ignore auditory stimulation, thereby reducing tinnitus. We speculate that as a result, the functional connection between the precuneus and the auditory network in the tinnitus group may decrease.

However, in contrast to our hypothesis, we found no significant functional connection changes between the precuneus and the auditory network, while the left inferior occipital gyrus, left calcarine cortex, and left superior frontal gyrus showed less functional connection with the left precuneus in the tinnitus group than in the control group. The left superior frontal gyrus belongs to the FEF, which is involved in the regulation of eye movement, visual orientation, and other functions. The left inferior occipital gyrus and the left calcarine cortex are located in the visual cortex and play a role in the perception and processing of visual stimulation. Schmidt et al. [23] found that the connectivity between the FEF and left precuneus was decreased in tinnitus patients and this decrease was not responsible for hearing loss, which may be due to the influence of abnormal tinnitus sounds. This change indicates that the neural network connection in tinnitus patients is not sufficiently unobstructed and that this obstruction affects their processing of visual information. Braga et al. [32] found a correlation between eye movement and auditory attention. Even in the absence of visual stimulation, there is a decrease in eye movement when processing auditory stimulation. We speculate that the decrease in functional connectivity between the left precuneus and the left superior frontal gyrus is due to tinnitus. As Burton et al.'s research shows [7], there is a relationship between auditory and visual network activity. When the brain is forced to process the auditory stimulation of tinnitus, the activity of the visual network is inhibited. Our results show a decrease in the functional connection between the precuneus and visual network. There was no significant change related to the auditory network, which may be the result of the attempts by tinnitus patients to suppress attention to auditory hallucinations.

This study has several limitations. First, our sample size was not large enough, and therefore, the results were only experimental. However, our final results are still reliable because they passed multiple comparison corrections. Second, we did not group the patients according to the duration or severity of tinnitus. Thus, whether the FC of the DAN in tinnitus patients changes due to the duration or severity of tinnitus needs to be further studied. Third, most obtained results were located in the left hemisphere. Whether this is related to the fact that all our subjects were right-handed remains to be further investigated. Fourth, this study is a cross-sectional study. Although our results show changes in functional connections related to the DAN in patients with tinnitus, the causal relationship remains to be studied. Therefore, a larger sample size and longitudinal follow-up studies from multiple perspectives should be carried out in the future.

## 5. Conclusions

Our study mainly found that the left superior parietal lobule showed abnormal functional connections within the DAN, while the left inferior parietal lobule and the left precuneus in the DAN showed abnormal activation in relation to the neural activity of the whole brain. We also found that the tinnitus patients exhibited different FC from the left superior parietal gyrus or the left precuneus to several brain regions belonging to the DAN, the limbic system, or some vision-related brain regions. These results may be used to reveal changes in neural activity in the brains of patients with tinnitus and may indicate that the DAN plays a significant role in the production and perception of tinnitus. For the treatment of tinnitus, the above results also offer some inspiration as follows: it may be possible to improve the disturbance and impact of tinnitus by regulating the function of the attention system, such as by reducing attention to tinnitus or diverting attention to other areas.

## Figures and Tables

**Table 1:** Demographic and clinical data of the tinnitus patients and healthy controls.

	TP (*n* = 31)	HC (*n* = 33)	*P* value
Age (years)	38.58 ± 13.20	38.24 ± 12.67	0.917^a^
Gender (male/female)	24/7	23/10	0.485^b^
Education^c^	9/8/8/6	6/11/6/10	0.370^b^
THI score	46.26 ± 26.26	NA	NA
Tinnitus duration (months)	32.68 ± 55.54	NA	NA

Data are presented as the mean ± standard deviation. ^a^These variables were compared by using two-sample *t*-tests. ^b^These variables were compared by using chi-square tests. ^c^The education level of all subjects was divided into the following categories: junior high school/senior high school or special secondary school/junior college/bachelor's degree or above. Acronyms: NA: not applicable; TP: tinnitus patients; HC: healthy controls; THI: Tinnitus Handicap Inventory.

## Data Availability

The datasets used and analyzed in the current study are available from the corresponding author upon reasonable request.
